# Surgical treatment strategies for patients with type A aortic dissection involving arch anomalies

**DOI:** 10.3389/fcvm.2022.979431

**Published:** 2022-09-13

**Authors:** Jiade Zhu, Guang Tong, Donglin Zhuang, Yongchao Yang, Zhichao Liang, Yaorong Liu, Changjiang Yu, Zhen Zhang, ZeRui Chen, Jie Liu, Jue Yang, Xin Li, Ruixin Fan, Tucheng Sun, Jinlin Wu

**Affiliations:** Department of Cardiac Surgery, Guangdong Cardiovascular Institute, Guangdong Provincial People's Hospital, Guangdong Academy of Medical Sciences, Guangzhou, China

**Keywords:** aberrant right subclavian artery (ARSA), isolated left vertebral artery (ILVA), surgical procedures, arch anomalies, cannulation and perfusion, hybrid therapy, total arch replacement, bovine arch

## Abstract

**Objective:**

The aim of the study was to investigate surgical modalities and outcomes in patients with type A aortic dissection involving arch anomalies.

**Method:**

Patients with type A aortic dissection who underwent surgical treatment at our center between January 2017 and 31 December 2020 were selected for this retrospective analysis. Data including computed tomography (CT), surgical records, and cardiopulmonary bypass records were analyzed. Perioperatively survived patients were followed up, and long-term mortality and aortic re-interventions were recorded.

**Result:**

A total of 81 patients with arch anomalies were included, 35 with “bovine” anomalies, 23 with an aberrant right subclavian artery, 22 with an isolated left vertebral artery, and one with a right-sided arch + aberrant left subclavian artery. The strategies of arch management and cannulation differed according to the anatomic variation of the aortic arch. In total, seven patients (9%) died after surgery. Patients with “bovine” anomalies had a higher perioperative mortality rate (14%) and incidence of neurological complications (16%). Overall, four patients died during the follow-up period, with a 6-year survival rate of 94.6% (70/74). A total of four patients underwent aortic re-intervention during the follow-up period; before the re-intervention, three received the *en bloc* technique (13.6% 3/22) and one received hybrid therapy (11.1% 1/9).

**Conclusion:**

With complete preservation and reconstruction of the supra-arch vessels, patients with type A aortic dissection combining arch anomalies can achieve a favorable perioperative prognostic outcome. Patients who received the *en bloc* technique are more likely to require aortic re-intervention than patients who underwent total arch replacement with a four-branched graft vessel. Cannulation strategies should be tailored according to the variation of anatomy, but routine cannulation with the right axillary artery can still be performed in most patients with arch anomalies, even for patients with an aberrant right subclavian artery.

## Introduction

Surgical management of type A aortic dissection requires close attention to the presence of aortic arch anomalies for timely adjustment of surgical treatment strategies. Anatomic abnormalities of the aortic arch are widely present in the general population. According to previous studies, 15–25% of the population may carry aortic arch anomalies (1–3). However, case reports documenting successful treatment of aortic dissection in the setting of anatomic anomalies of the aortic arch are sporadic and with a small sample size (2, 4–12).

The presence of arch anomalies cannot always be treated using routine cannulation strategies; for instance, patients with an ARSA usually cannot undergo selective cerebral perfusion (SCP) through the right axillary artery, which is a routine SCP cannulation location. On the other hand, the arch technique differs much in patients with arch anomalies from the standard procedure.

In this study, we reported our experience in managing type A aortic dissection involving arch anomalies, focusing on surgical techniques, postoperative outcomes, and follow-up results. In addition, our surgical strategies for four major types of arch anomalies were described in detail, and the surgical details and indications of specific procedures have been fully discussed to provide a comprehensive reference for selecting surgical strategies for these patients.

## Materials and methods

### Patients and data collection

**Figure 1:**
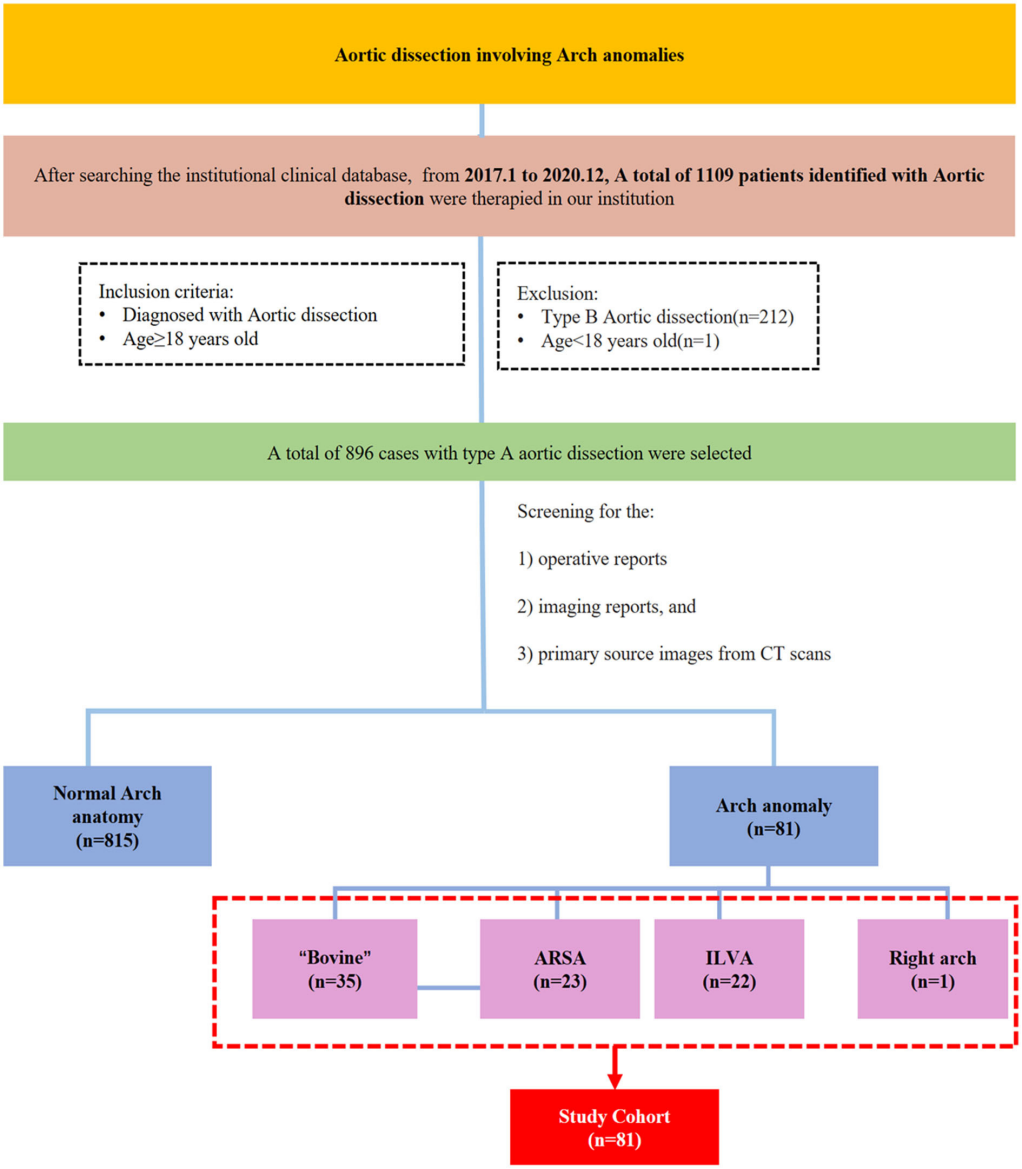
Study flowchart.

Patients with type A aortic dissection who underwent surgical treatment from January 2017 to 31 December 2020 at our center were selected for this retrospective analysis. A total of 81 patients were diagnosed with arch anomalies by computed tomography (CT) reports or based on surgical records. The study flowchart, including the inclusion and exclusion criteria, is shown in Figure 1.

The hospital ethics committee has approved this research (GDREC2018322H).

### Data collection and follow-up

Clinical data were collected from all included patients. Operative records, anesthesia records, and perfusion records were used to determine operative variables, including cannulation strategy, method of surgical repair, site of primary intimal tear (PIT), bypass times, nadir temperature, use of hypothermia and circulation arrest (HCA), and selected cerebral perfusion (SCP). Progress notes and discharge summaries were used to determine the incidence of postoperative complications and mortality. In addition, demographic records, imaging reports, and primary source images from CT scans were collected and analyzed.

The follow-up was carried out over telephone to the latest time to find out the mid-term survival and the incidence of aortic re-intervention. For patients with positive outcomes, the follow-up interval was calculated until the point the positive outcome occurred.

### Diagnose arch anomalies

Arch anomaly types were identified using details from the operative reports, imaging reports, and primary source images from CT scans. A total of four major types of abnormal arch anatomy were identified: (1) “bovine” anomaly: an arch with a common origin of the innominate artery (IA) and left common carotid artery (LCCA), or the LCCA originating directly from the IA; (2) aberrant right subclavian artery (ARSA): a four-vessel arch with an aberrant right subclavian artery originating from the distal arch or proximal descending aorta; (3) isolated left vertebral artery (ILVA): a four-vessel arch with the left vertebral artery coming directly from the aorta; (4) right arch with the aberrant left subclavian artery (ALSA): a mirror image to the ARSA, a four-vessel aortic arch is in the right side of the main trachea, with an ALSA originating from the distal arch or proximal descending aorta.

### Surgical techniques

All procedures were performed under general anesthesia and cerebral flow monitoring using cerebral oximetry. Patients who underwent total arch replacement (TAR) or right hemi-arch replacement had HCA and SCP.

**Figure 2:**
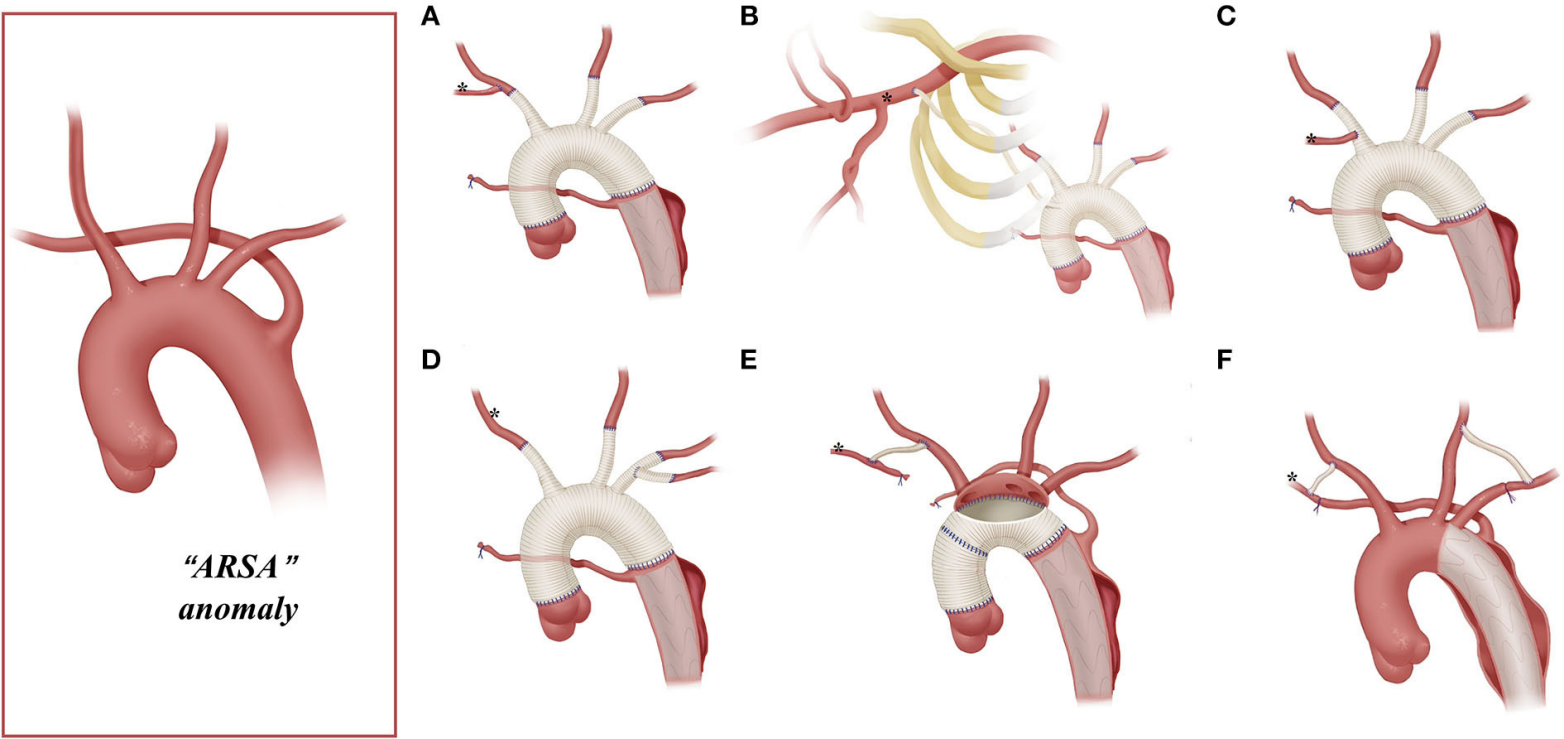
Arch technique for ARSA in patients with type A aortic dissection. *Distal ARSA; **(A)** stage TAR for ARSA; **(B)** extra-anatomy revascularization of the right axillary artery; **(C)** ARSA connected to the vascular graft of the RCCA; **(D)** ARSA directly anastomosed to the 8-mm arch branch of the four-branched graft; **(E)** *en bloc* technique for the ARSA; **(F)** hybrid technique for ARSA.

A total of three techniques were used for arch reconstruction: TAR, right hemi-arch replacement, and hybrid technique. The TAR technique included TAR with a four-branched graft vessel and TAR with the *en bloc* technique. TAR with a four-branched graft vessel has been described (13, 14) previously. A four-branched graft was used in TAR combined with stented elephant trunk (SET) implantation under the condition of HCA and SCP. The *en bloc* technique was described by Zhong et al. (15). The anterior wall of the aortic arch was incised longitudinally up to the origin of the left common carotid artery (LCCA); no dissection of the arch vessels was confirmed intraoperatively. After deployment of the SET, a balloon was implanted inside the SET to recover the femoral cannula perfusion. Then, the stent-free sewing edge (3- to 5-cm-long Dacron graft) of the SET was straightened and trimmed. Next, under the condition of SCP and femoral perfusion, the trimmed sewing edge was sutured to the native aortic wall near the origins of the arch branches (shown in Figure 2E).

Right hemi-arch replacement was performed as follows: the lesser curvature of the aortic arch was incised and replaced with a single Dacron graft vessel.

The hybrid technique was performed as follows: a GORE-TEX vascular graft was used for revascularization of the supra-arch vessels using incisions in the cervical and supraclavicular fossa regions according to the anatomy feature. Then, a thoracic stent graft was deployed retrograde *via* the femoral artery access. Angiography was performed to confirm the deployment position and GORE-TEX graft patency.

The detailed steps of the reconstruction techniques are described in the Supplementary material.

**Figure 3:**
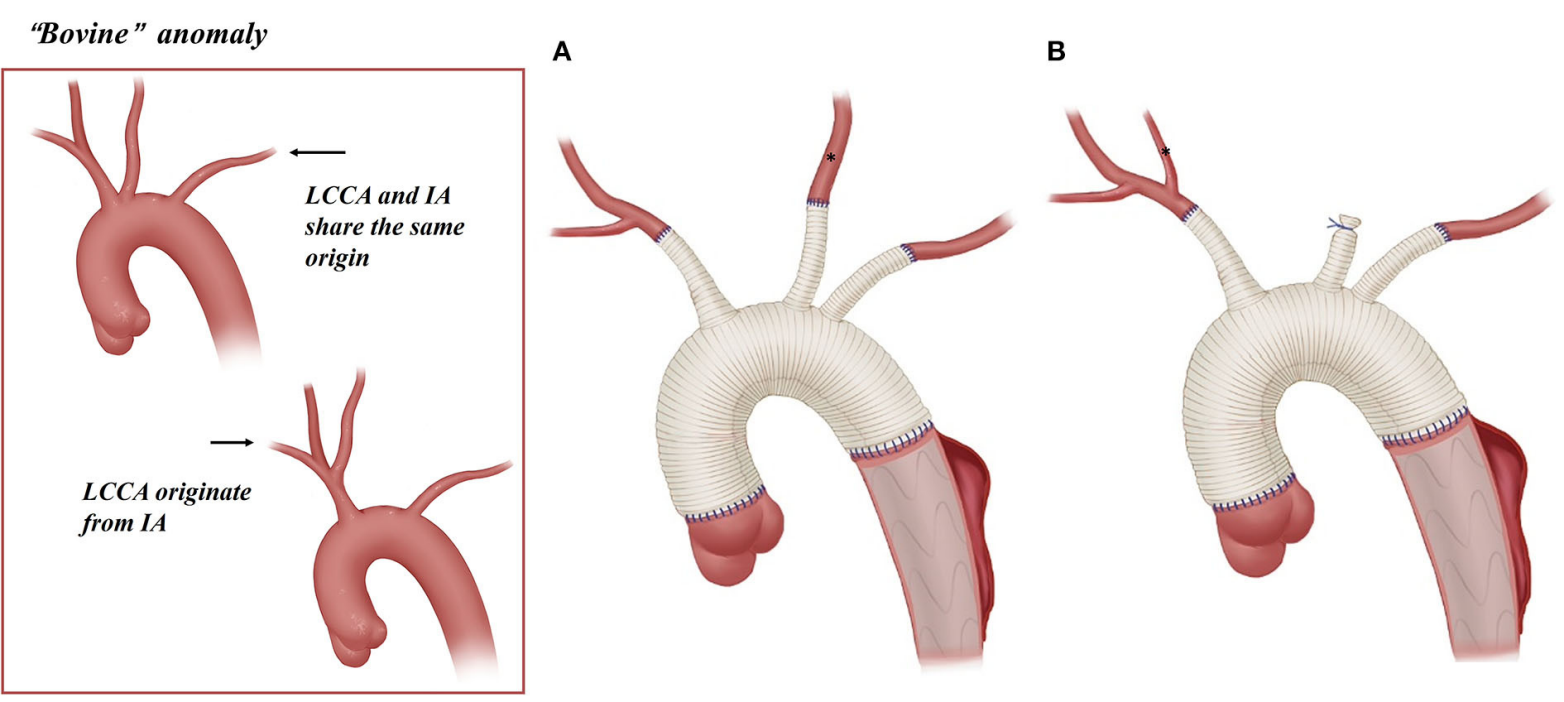
Arch technique for “bovine” anomaly in patients with type A aortic dissection. *LCCA; **(A)** reconstruction with three graft branches for “bovine” anomaly. **(B)** Reconstruction with two graft branches for “bovine” anomaly.

**Figure 4:**
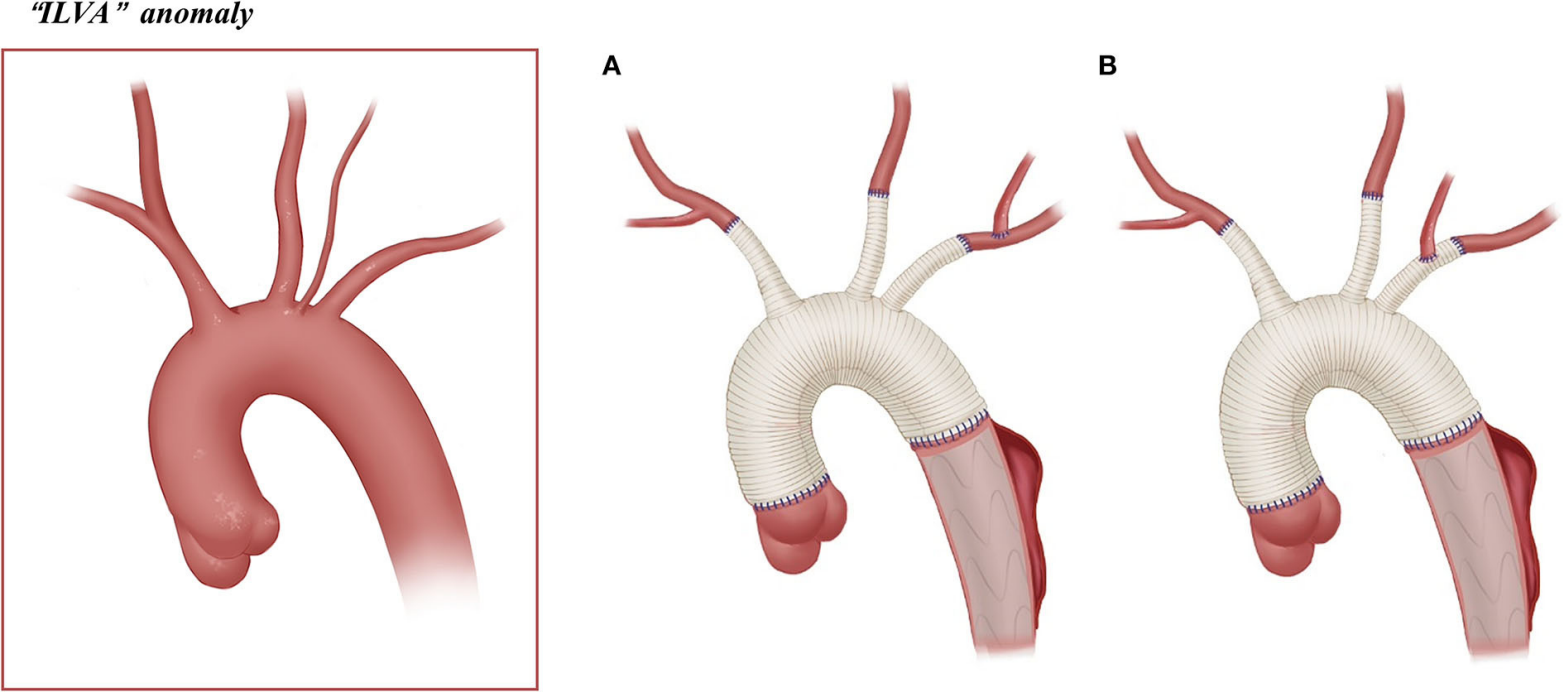
Arch technique for “ILVA” anomaly in patients with type A aortic dissection. **(A)** ILVA anastomosed to the LSA. **(B)** ILVA anastomosed to the LSA graft branch.

**Figure 5:**
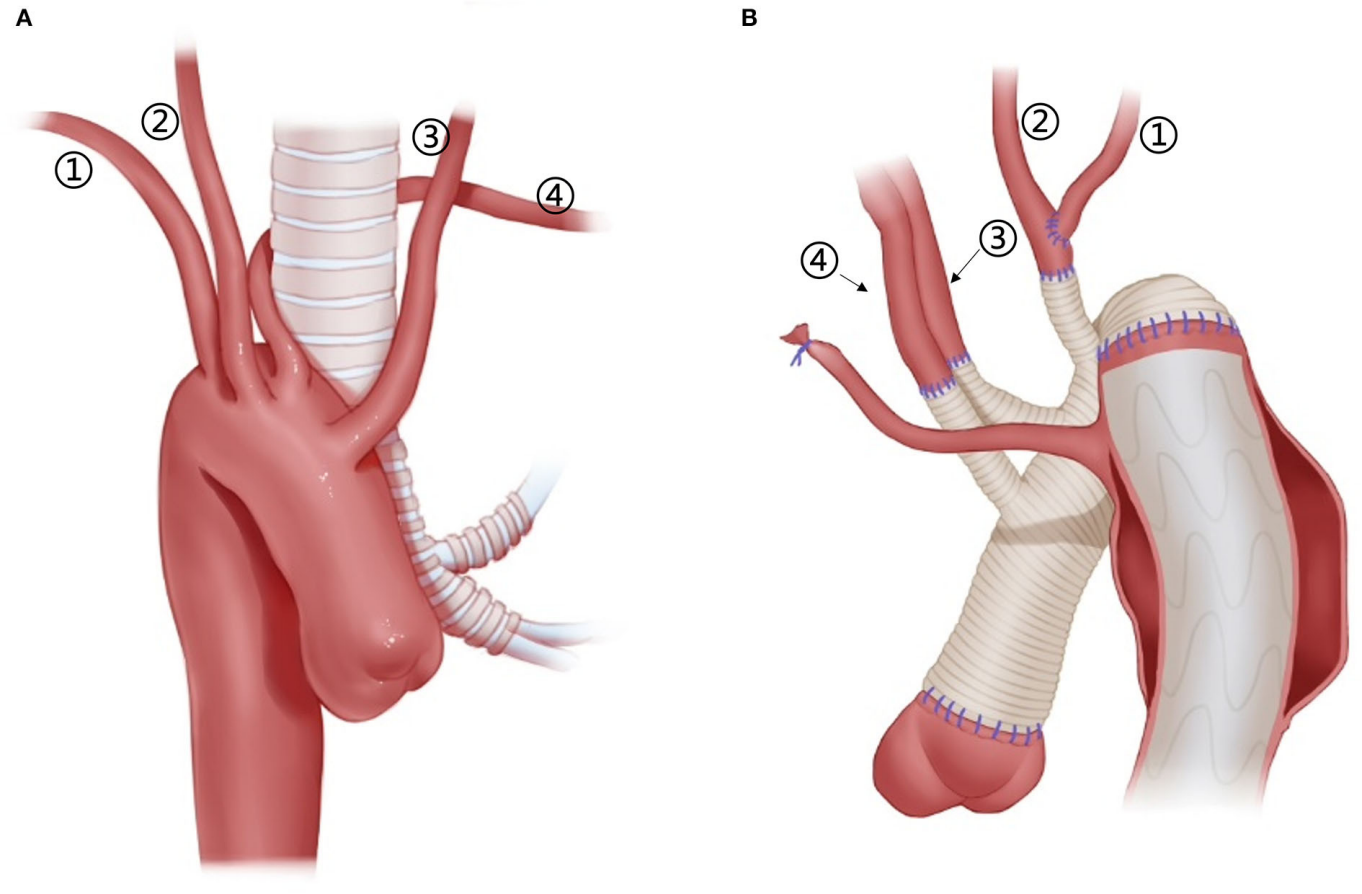
Arch technique for patients with right arch and ALSA. (1) RSA; (2) RCCA; (3) LCCA; (4) ALSA. **(A)** Schematic diagram of the anatomy of the right arch + ALSA, from the front view. **(B)** Arch technique for a patient with right arch + ALSA, from the posterior view. ALSA, from the posterior view.

The arch management strategies of the 81 included patients are summarized in Table 1. Figures 2–5 are the schematic diagrams of the arch management strategies for patients with an ARSA, bovine anomaly, ILVA, and right arch combining the ALSA, respectively.

**Table 1:** Arch management strategies for different aortic arch deformities.

	***TAR***		**Right hemi-arch replacement**	**Hybrid technique**
	**TAR with four-branched graft**	***En* bloc**		
**ARSA**, ***n*** **=** **23**	*2 (2-A)+ 5 (2-B)+ 3 (2-C) +2 (2-D)	4(2-E)		7 (2-F)
**Bovine**, ***n*** **=** **35**	10 (3-A) +8 (3-B)	14	2	1
**ILVA**, ***n*** **=** **22**	7 (4-A)+10 (4-B)	4		1
**Right arch**, ***n*** **=** **1**	1 (5-B )			

The context within parentheses represents the specific arch techniques that were used, and the numbers before the parentheses represent the cases of the corresponding method. For example, ^*^represents two patients with an ARSA who underwent the arch technique shown in Figure 2A.

Among patients with an ARSA who underwent TAR with a four-branched graft, two patients underwent two-stage TAR. First, the ARSA was fully mobilized and anastomosed to the RCCA end-to-side to recover a normal anatomy structure, and then the CPB and SCP were performed with the routine cannulation of the right axillary artery (Figure 2A).

A total of five patients with an ARSA underwent extra-anatomic revascularization of the ARSA (Figure 2B). After the coronary perfusion was restored, the heart resumed beating, and the fourth perfusion side arm of the four-branched graft was anastomosed to the right axillary artery in an end-to-side fashion through the right thoracic cavity and the second intercostal space.

A total of 22 patients underwent the *en bloc* technique, among which four patients with an ARSA needed extra-RCCA-to-distal ARSA bypass using the cervical and right supraclavicular fossa incisions; two patients (“bovine” anomaly) underwent right hemi-arch replacement using a single Dacron graft vessel, which was anastomosed to the trimmed margin of the lesser curvature of the aortic arch in an end-to-end fashion with running stitches of 5-0 polypropylene.

A total of nine patients underwent hybrid therapy (ARSA in seven, “bovine” in one, and ILVA in one), and common carotid artery-to- subclavian artery bypass using the cervical and supraclavicular fossa incisions with a 7-mm GORE-TEX graft was accomplished before the implantation of the thoracic stent graft.

### Statistical analysis

Continuous variables were expressed as mean ± standard deviation or median (0.25–0.75 interquartile). Kolmogorov–Smirnov analysis was used to clarify whether data conformed to normal distribution. Categorical variables were expressed as the number of cases (percentage), for example, 28 (80%), indicating 28 cases occupying 80% of the cases of this group. Figures were drawn using Microsoft PowerPoint 2019 (Microsoft, Redmond, USA) or Easy Paint Tool SAI (SYSTEMAX, Japan) software. A *p* < 0.05 was considered statistically significant.

## Results

A total of 896 patients with type A aortic dissection were treated in our center from January 2016 to December 2020. After screening the CT reports and surgical records, 81 patients with arch anomalies were included in this study; 35 had “bovine” anomaly, 23 had an ARSA, 22 had an ILVA, and right-sided arch combined with an ALSA was found in one case (Figure 1).

### Baseline data

Baseline data of the 81 patients are shown in Table 2. Overall, 73% of the patients had hypertension, and 45 (56%) had hyperlipidemia; seven (9%) patients had coronary malperfusion. The incidence of other malperfusion syndromes was given as follows: neurological ischemia (14%), upper extremity ischemia (5%), spinal ischemia (2%), mesenteric ischemia (20%), renal ischemia (23%), and lower extremity ischemia (10%).

**Table 2:** Baseline characteristics.

	**Bovine arch (n = 35)**	**ARSA (n = 23)**	**ILVA (n = 22)**	**Right Arch (n = 1)**	**All (*n* = 81)**
Age	49.5 ± 10.9	47.4 ± 11.7	52.6 ± 9.7	48	49.7 ± 10.8
Male gender	28 (80%)	17 (74%)	20 (91%)	1 (100%)	66 (81%)
BMI	23.6 ± 4.0	26.0 ± 4.7	24.5 ± 3.0	24.6	24.8 ± 4.1
**Malperfusion syndrome**					
Cardiac ischemia	6 (17%)	0	1 (5%)	0	7 (9%)
Neurological deficit	6 (17%)	3 (13%)	2 (9%)	0	11 (14%)
Upper extremities ischemia	1 (3%)	2 (8%)	1 (5%)	0	4 (5%)
Spinal ischemia	1 (3%)	0	1 (5%)	0	2 (2%)
Mesenteric ischemia	8 (23%)	3 (13%)	5 (23%)	0	16 (20%)
Renal ischemia	8 (23%)	5 (22%)	6 (27%)	0	19 (23%)
Lower extremities ischemia	7 (20%)	0	1 (5%)	0	8 (10%)
**Dissection type**					
Debakey I	28 (80%)	13 (57%)	16 (73%)	0	57 (70%)
Debakey II	4 (11%)	0	2 (9%)	0	6 (7%)
None - A None - B	3 (9%)	10(43%)	4 (18%)	1(100%)	18(22%)
AI (more than moderate)	14 (40%)	5 (22%)	8 (36%)	0	27 (33%)
Hypertension	23 (66%)	18 (78%)	17 (77%)	1(100%)	59 (73%)
Diabetes mellitus	0	1 (4%)	1 (5%)	0	2 (2%)
Hyperlipidemia	21 (60%)	14 (61%)	10 (46%)	0	45 (56%)
CAD	11 (31%)	7 (30%)	5 (23%)	0	23 (28%)
Marfan	0	2 (9%)	0	0	2 (2%)
BAV	1 (3%)	0	0	0	1 (1%)
History of cardiac surgery	1 (3%)	1 (4%)	1(5%)	0	3 (4%)
History of TEVAR/EVAR	0	2 (9%)	1(5%)	0	3 (4%)

Corresponding percentages are given within parentheses.

In all, one patient with “bovine” anomaly had a bicuspid aortic valve (3%); three patients underwent cardiac surgery (one in the “bovine” anomaly, one in the ARSA, and one in the ILVA), and two patients with an ARSA and one patient with an ILVA received implantation of the stent graft in the thoracic/abdominal aorta before admission to our department.

### Surgical data

The surgical data of the 81 patients are summarized in Table 3. The location of PIT was explored intraoperatively: aortic root or ascending aorta in 48%, aortic arch in 37%, descending aorta in 12%, and no PIT in two cases. All patients who underwent TAR or right hemi-arch replacement had HCA+antegrade SCP. The nasopharyngeal temperature was reduced to 20–25°C during HCA. Other concomitant procedures included three cases of coronary artery bypass grafting (two in the “bovine” anomaly and one in the ILVA), one case of left atrial thrombosis removal (ARSA), one case of mitral + tricuspid valvuloplasty (“bovine”), and one case of the Nuss procedure (“bovine”).

**Table 3:** Surgical characteristics.

	**Bovine arch (*n* = 35)**	**ARSA (*n* = 23)**	**ILVA (*n* = 22)**	**Right arch (*n* = 1)**	**All (*n* = 81)**
**Site of PIT**					
Root/Ascending	20 (58%)	10 (43%)	9 (41%)	0	39 (48%)
Aortic arch	11 (31%)	8 (35%)	10 (46%)	1 (100%)	30 (37%)
Descending	14 (11%)	4 (17%)	2 (9%)	0	10 (12%)
None	0	1 (4%)	1 (5%)	0	2 (2%)
**Cases with CPB**	**34 (97**%**)**	**16 (70**%**)**	**21 (95**%**)**	1 (100%)	**72 (89**%**)**
**Arterial bypass cannulation**					
Axillary*	16 (46%)	4 (17%)	12 (55%)	0	32 (40%)
Femoral	3 (9%)	9 (39%)	5 (23%)	0	17 (21%)
Axillary+Femoral	9 (26%)	0	2 (9%)	0	11 (14%)
Innominate+Femoral	5 (14%)	2 (9%)	2 (9%)	1 (100%) **	10 (12%)
Aortic arch	1 (3%)	1 (4%)	0	0	2 (2%)
**Cases with HCA and SCP**	**34 (97**%**)**	**16 (70**%**)**	**21 (95**%**)**	1 (100%)	**72 (89**%**)**
RUACP	19 (54%)	5 (22%)	10 (46%)	1 (100%)	35 (43%)
LUACP	2 (6%)	6 (26%)	5 (23%)	0	12 (15%)
BiACP	13 (38%)	5 (22%)	6 (27%)	0	24 (30%)
CA time	21.9 ± 9.2	25.3 ± 9.2	25.5 ± 8.9	26	23.8 ± 9.1
Nadir temperature	23.2 ± 3.9	22.4 ± 3.9	21.8 ± 2.3	19.8	22.5 ± 3.5
**Hybrid procedure**	1 (3%)	7 (30%)	1 (5%)	0	9 (11%)

* Axillary or innominate, ^**^RCCA + femoral. Corresponding percentages are given within parentheses. PIT, primary intimal tear; RUACP, right unilateral antegrade cerebral perfusion; LUACP, left unilateral antegrade cerebral perfusion; BiACP, bilateral antegrade cerebral perfusion.

Among 34 patients with “bovine” anomaly who underwent CPB, 30 patients (88%, 30/34) received CPB through the cannulation of a right axillary artery or innominate artery, and 14 received CPB with combined cannulation of a right axillary/IA and femoral artery. A total of 13 patients (38%) with “bovine” anomaly achieved BiACP by cross-clamping of the common origin of the IA and LCCA and cannulation of the right axillary artery/common origin, and 19 patients underwent RUCP with cross-clamp applied to the cephalad to the origin of the LCCA and cannulation of the right axillary artery.

CPB was performed in 16 of 23 patients with an ARSA; 11 patients underwent CPB through the cannulation of the femoral artery (69%, 11/16) and one patient through the cannulation of the aortic arch.

Among 22 patients with an ILVA, 21 underwent SCP. The SCP strategy in these patients is as follows: BiACP (*n* = 6) and RUACP (*n* = 10) were achieved through cannulation of the right axillary artery/IA with/without the LCCA, and LUACP (*n* = 5) was achieved through the cannulation of the LCCA.

Only one patient with a right-sided arch combining the ALSA was first operated with femoral artery cannulation for CPB and then RCCA cannulation for SCP.

### Surgical results

Perioperative outcomes are summarized in Table 4. Overall, seven patients (9%) died during postoperative hospitalization, and two patients required ECMO assistance (one ILVA and one “bovine”). Other postoperative complications are as follows: neurological complications (16%), dialysis treatment (17%), paraplegia (5%), and redo of tracheotomy (2%). Among them, 26% of patients with “bovine” anomaly had neurological complications, and all patients complicated with paraplegia had “bovine” anomaly.

**Table 4:** Surgical results.

	**Bovine arch**	**ARSA**	**ILVA**	**Right arch**	**All**
Early mortality	5 (14%)	1 (4%)	1 (5%)	0	7 (9%)
Re-exploration	3 (9%)	0	2 (9%)	0	5 (6%)
ECMO	1 (3%)	0	1 (5%)	0	2 (2%)
Neurologic*	9 (26%)	3 (13%)	1 (5%)	0	13 (16%)
Tracheotomy	2 (6%)	0	0	0	2 (2%)
Paraplegia	4 (11%)	0	0	0	4 (5%)
Dialysis	8 (23%)	3 (13%)	3 (14%)	0	14 (17%)

^*^Stroke, delirium, paresis, and paraplegia. Corresponding percentages are given within parentheses.

### Follow-up results

A total of 74 patients who survived perioperatively were followed up by telephone, with a follow-up rate of 100%. The mean follow-up interval was 36.8 months for long-term mortality, and 35.2 months for aortic re-intervention.

In total, four patients underwent aortic re-intervention during the follow-up period: three patients with the *en bloc* technique, among whom one underwent IA stenting for IA dissection 18 months postoperatively, one underwent intervention therapy for LCCA occlusion 6 months postoperatively, and one underwent intervention therapy for basilar artery dissection 8 months later; one patient with hybrid therapy underwent stainless steel coil implantation for stent endoleak 3 months later. A total of four patients died during the follow-up period, with a survival rate of 94.6% (70/74) at 6 years postoperatively.

## Discussion

Reports on treating patients with type A aortic dissection involving arch anomalies are rare. Our study is probably the largest cohort study in this field to date; we searched PubMed for English language articles reporting on aortic dissection and aortic arch malformation published in the past 10 years; studies with a sample size of more than 10 are summarized in Table 5.

**Table 5:** Major articles of aortic dissection involving arch anomalies.

**References**	**Country**	**Year**	**Cases**	**Types of arch anomalies**	**Stanford**	**Operative procedure**	**Journal**
Bryan et al. (5)	U.S	2001–2011	43	ARSA (5), bovine (32), right arch (3), ILVA (3)	A	Opening graft replacement	J Cardiothorac Vasc Anesth 2014
Sherene et al. (2)	U.S, Ireland	1990–2014	75	ARSA, bovine, right arch, ILVA	B	Not mentioned	J Vasc Surg 2018
Zhou et al. (6)	China	2010–2015	13	ARSA	B	Total Endovascular Treatment	Eur J Vasc Endovasc Surg 2017
Ding et al. (7)	China	2011–2016	16	ARSA	B	TEVAR and extra anatomic bypass hybrid procedure	J Vasc Surg 2018
					Non-A, Non-B		
Li et al. (4)	China	2009–2017	22	ARSA	A (15)	TAR + SET	Eur J Cardiothorac Surg 2020
					Non-A Non-B (7)		
Zhang et al. (8)	China	2012–2018	15	ARSA	B	Endovascular repair	J Vasc Interv Radiol 2019
Dumfarth et al. (9)	U.S., Austria	2002–2013	22	Bovine;	A	Opening graft replacement (22); TEVAR (2)	Ann Thorac Surg 2014
Zuo et al. (10)	China	2017–2019	13	ILVA	A	ILVA-LCCA bypass + TAR + SET	Eur J Cardiothorac Surg 2021
Ding et al. (11)	China	2011–2018	31	ILVA	B	TEVAR	J Vasc Surg 2019
Qi et al. (12)	China	2003–2008	21	ILVA	A (20), B (1)	TAR	Ann Thorac Surg 2013

The right axillary artery cannulation for CPB and SCP is a standard procedure for type A aortic dissection. In this study, this routine cannulation strategy was applicable for most patients with “bovine” anomaly and ILVA (“bovine,” 24/34; ILVA, 14/21), while it was not suitable for patients with an ARSA due to the unique anatomic structures found in these patients. In our group, only two patients with an ARSA underwent the standard cannulation procedure, and their ARSA was mobilized, dissected, and anastomosed to RCCA before proceeding to the standard cannulation procedure (Figure 2A).

This stage TAR technique requires complete mobilization of the ARSA and should ensure that dissection does not involve the RCCA and the distal part of the ARSA. However, for some patients, mobilizing the ARSA may be challenging. In addition, the ARSA might be involved by dissection, rendering the establishment rather difficult for a near-normal anatomic ARSA-to-RCCA connection in advance. In this study, 75% (12/16) of the patients with the ARSA underwent CPB *via* cannulation of the femoral artery or aortic arch, instead of the right axillary artery.

Unilateral or bilateral cerebral perfusion can be achieved by cannulation in the right axillary artery, common carotid artery, or IA. Patients with unilateral cerebral perfusion require full consideration of the integrity of the circle of Willis. BiACP is usually required in case of an incomplete circle of Willis or significant asymmetry between left and right cerebral oxygen saturation. In our clinical practice, we found that the SCP strategy in patients with arch anomalies differs from the conventional practice. For patients with “bovine” anomalies, the cerebral perfusion strategy may change depending on the location of the origin of the LCCA. In cases where the origin of the LCCA is located high in the IA, BiACP can be achieved with a clamp applied caudad to the IA and cannulation of the right axillary artery. Bryan et al. (5) described this strategy in 11 patients with “bovine” anomaly. In our cohort, 31% of patients with “bovine” anomaly underwent BiACP, which is significantly higher than other anomaly groups. Patients with the ARSA cannot undergo RUSCP *via* the cannulation of the right axillary artery [except for patients who underwent two-stage TAR (16), *n* = 2, Figure 2A]. As a result, SCP can only be performed with cannulation of the LCCA and/or RCCA for these patients. For patients with an ILVA, additional cannulation of the ILVA for cerebral perfusion is not needed. Qi et al. (12) treated 21 ILVA patients without additional cannulation of the ILVA; only two (9.5%, 2/21) patients had neurological complications after the surgery. This is consistent with the results in this cohort, in which 22 patients with an ILVA were treated without additional cannulation in the ILVA, and their neurological complication rate was even lower than that of the ARSA or “bovine” groups (incidence of neurological complications was 26, 13, and 5% for “bovine,” ARSA, and ILVA, respectively).

Arch reconstruction with preservation of all the supra-arch vessels is recommended for patients with arch anomalies. The *en bloc* technique is a simple strategy that can be computed within a relatively short time, with a single aortic patch containing the origin of all the supra-arch vessels anastomosed to the stent-free sewing edge of SET. Yet, according to the follow-up result in this study, these patients may have a higher incidence of aortic re-intervention; the reason for this might include (1) potential dissection in the supra-arch vessels not found intra-operatively, or (2) intimal tears during the cannulation and clamping of the supra-arch vessels that could lead to potential dissection or stenosis. Patients who underwent TAR with a four-branched graft had a better follow-up result with no aortic re-intervention event. The side arms of the four-branched graft were attached to the supra-arch vessels. For patients with “bovine” anomaly, revascularization with only two side arms of the four-branched graft can be achieved (Figure 3B) in cases whose LCCA directly originates from the IA. For patients with an ILVA, the ILVA should be preserved and anastomosed to the LSA (Figure 4A) or its side arm (Figure 4B). Preservation of the ILVA can guarantee the integrity of the circle of Willis and reduce neurological complications. Qi et al. (12) reported another technique for the preservation of the ILVA, with “*a single aortic patch containing the origin of the ILVA and LSA anastomosed to one limb of the prosthetic graft*.” For patients with an ARSA, if the ARSA is involved by dissection or is difficult to mobilize, extra-anatomic reconstruction can be performed with the fourth perfusion side arm (Figure 2B). Another extra-anatomic revascularization method might be the RCCA-to-ARSA bypass through cervical + right subclavian incisions, which is not recommended in our center as it can damage venous plexuses in the subcutaneous tunnel, resulting in massive venous hemorrhage. It may also compress the shunt graft and lead to distant occlusion. By comparison, the extra-anatomic revascularization through the right thoracic cavity (Figure 2B) can avoid the compression by the subcutaneous tunnel without extra-cervical incision, and no shunt graft obstruction was found during the follow-up period in our cohort, indicating its safety and stability.

A hybrid technique has been reported in several studies as a treatment for patients with type B aortic dissection involving the ARSA and ILVA (6–8, 11). In this cohort, the hybrid technique was used for patients with type non-A non-B aortic dissection whose aortic arch was minimally involved (50%, 9/18). For patients with a severely compromised aortic arch and those for whom complete revascularization of the supra-arch vessels cannot be ensured after TEVAR implantation (e.g., patients with RCCA or IA involvement), hybrid therapy should not be recommended. For patients with an ARSA undergoing a hybrid technique, the distal part of the ARSA can always be found in the right supraclavicular fossa area. After completing the supra-arch bypass, the proximal part of the subclavian arteries will be clamped tentatively and then ligated if there is no significant change in blood pressure in the upper extremity. First, tentative clamping of the subclavian arteries is necessary to ensure the common carotid artery-to-subclavian artery bypass is adequate for the perfusion of the upper extremities. Second, the proximal part of the ARSA and LSA should better be ligated to reduce the occurrence of competing for blood flow and type II endoleak. Third, supra-arch vessels bypassing the subcutaneous tunnel by using cervical + subclavian incisions are not recommended. The reason for this has been discussed before. The stent graft should be positioned carefully before release because it might occlude the origin of the ILVA and some other supra-arch vessels, especially in patients whose ILVA originates from the distal arch. Our interventional team has performed TEVARs in 31 patients with type B aortic dissection involving the ILVA anomaly. Before the release of the stent graft, angiography was performed to confirm that the distal part of the stent graft is at least 1 cm away from the take-off of the ILVA, and no blocking of the ILVA was observed (11).

The distal management strategy mainly included the implantation of the SET or TEVAR stent. It is important to note that in patients with an ARSA, complete coverage of the ARSA origin with a SET or TEVAR stent is needed to isolate the Kommerell diverticulum (4). The follow-up results of this cohort showed only one patient with endoleak after TEVAR therapy; no endoleak was found in patients with SET implantation, demonstrating the effectiveness and stability of SET or TEVAR stent implantation.

The perioperative mortality rate (9%) in this cohort is somehow consistent with previous studies (14, 17, 18) that reported a perioperative mortality rate of 5–15% in patients with pure type A aortic dissection. This suggests that aortic arch malformation may not be a high risk for perioperative mortality for patients with type A aortic dissection (5, 9). Some studies suggested a higher incidence of neurological complications in patients with “bovine” anomalies (9), which is consistent with the results of this study: patients with “bovine” anomalies had a higher incidence of neurological complications and perioperative mortality than patients with other anomalies. The reason for this is not clear. Future studies with a larger sample size are needed to confirm the impact of specific aortic arch anomalies on the perioperative prognosis.

This study has a few limitations: (1) patients with type B aortic dissection were not included in this study; (2) this was a retrospective analysis; however, due to the urgency of type A aortic dissection, it is almost impossible to perform a prospective cohort analysis.

## Conclusion

For patients with type A aortic dissection combining arch anomalies, complete arch reconstruction with preservation of all the supra-arch vessels and reasonable cannulation strategies should be considered with an elaborate design based on the anatomical features so as to achieve a favorable perioperative and long-term prognosis outcomes. Routine cannulation with the right axillary artery can be achieved in most cases, even in patients with an ARSA. Moreover, patients who undergo the *en bloc* technique may have a higher risk of dissection or stenosis of the supra-arch vessels, and more of them need aortic re-intervention compared with patients who undergo TAR with a four-branched graft.

## Data availability statement

The raw data supporting the conclusions of this article will be made available by the authors, without undue reservation.

## Author contributions

JZ: primary manuscript writing, revision, design of conception, data collection, and literature search. GT, DZ, YY, ZL, YL, CY, and JY: data collection. ZZ, XL, and RF: provision of patients. ZC: data collection and provision of patients. JL: administrative support. TS: provision of patients, obtaining funding, final approval of the article, and administrative support. JW: manuscript revision, design of conception, data collection, and literature search. All authors contributed to the article and approved the submitted version.

## Funding

This work was supported by the National Key Research and Development Program of China (2018YFC1002600) and the Science and Technology Planning Project of Guangdong Province, China (2017A070701013, 2017B090904034, 2017B030314109, and 2019B020230003), the Guangdong Peak Project (DFJH201802), and Guangzhou Science and Technology Program Key Projects (202002020037) and Guangdong special funds for science and technology innovation strategy, China (Stability support for scientific research institutions affiliated to Guangdong Province-GDCI 2021).

## Conflict of interest

The authors declare that the research was conducted in the absence of any commercial or financial relationships that could be construed as a potential conflict of interest.

## Publisher's note

All claims expressed in this article are solely those of the authors and do not necessarily represent those of their affiliated organizations, or those of the publisher, the editors and the reviewers. Any product that may be evaluated in this article, or claim that may be made by its manufacturer, is not guaranteed or endorsed by the publisher.

## Supplementary material

Supplementary Figure S1 CT construction image after hybrid technique for ARSA. (1) Graft connecting LCCA and LSA. (2) Left common carotid artery. (3) Right common carotid artery. (4) Graft connecting RCCA and RSA. (5) Remnant of LSA. (6) Elephant trunk stent. (7) False lumen. (8) Distal aberrant right subclavian artery.

Supplementary Figure S2 CT images of the patient with right arch+ALSA. **(A)** Pre-operative CT construction image. **(B)** CT scan images. **(C)** Postoperative CT construction image. (1) ALSA; (2) LCCA; (3) RCCA; (4) False lumen; (5) Right arch; (6) Right-sided descending thoracic aorta; (7) RSA.

## Abbreviations

ALSA: aberrant left subclavian artery
ARSA: aberrant right subclavian artery
BiACP: bilateral antegrade cerebral perfusion
CT: computed tomography
HCA: hypothermia and circulation arrest
IA: innominate artery
ILVA: isolated left vertebral artery
LCCA: left common carotid artery
LSA: left subclavian artery
LUACP: left unilateral antegrade cerebral perfusion
PIT: primary intimal tear
RCCA: right common carotid artery
RSA: right subclavian artery
RUACP: right unilateral antegrade cerebral perfusion
SCP: selected cerebral perfusion
SET: stented elephant trunk
TAR: total arch replacement.
