# Supplementary figures and images for: Surgical treatment strategies for patients with type A aortic dissection involving arch anomalies
Source: Front Cardiovasc Med. 2022 Sep 13;9:979431. doi: 10.3389/fcvm.2022.979431 (PMC9513207; doi:10.3389/fcvm.2022.979431)

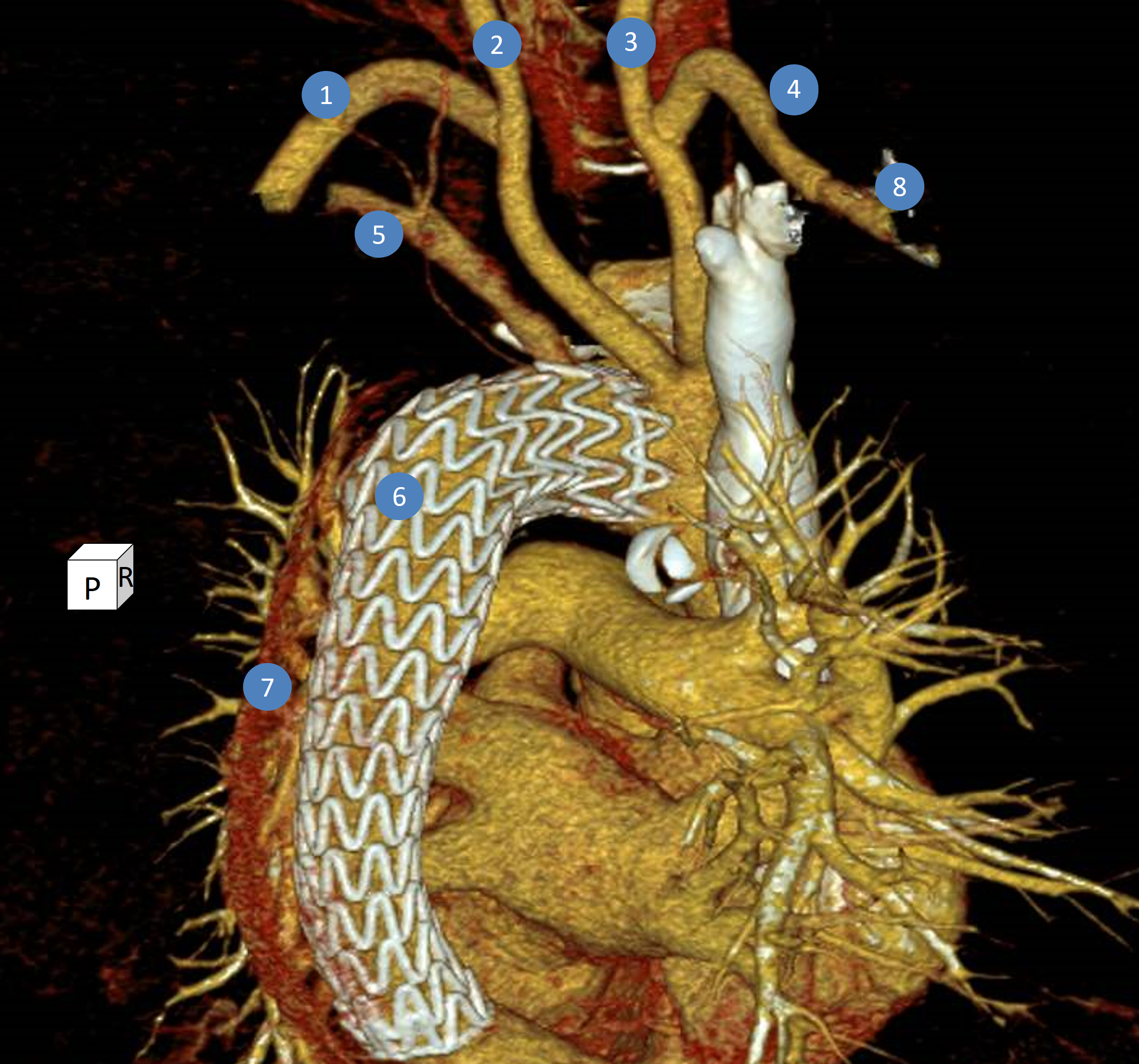

Supplement: Supplementary Figure S1 — CT construction image after hybrid technique for ARSA. (1) Graft connecting LCCA and LSA. (2) Left common carotid artery. (3) Right common carotid artery. (4) Graft connecting RCCA and RSA. (5) Remnant of LSA. (6) Elephant trunk stent. (7) False lumen. (8) Distal aberrant right subclavian artery. [file Image_1.TIF]

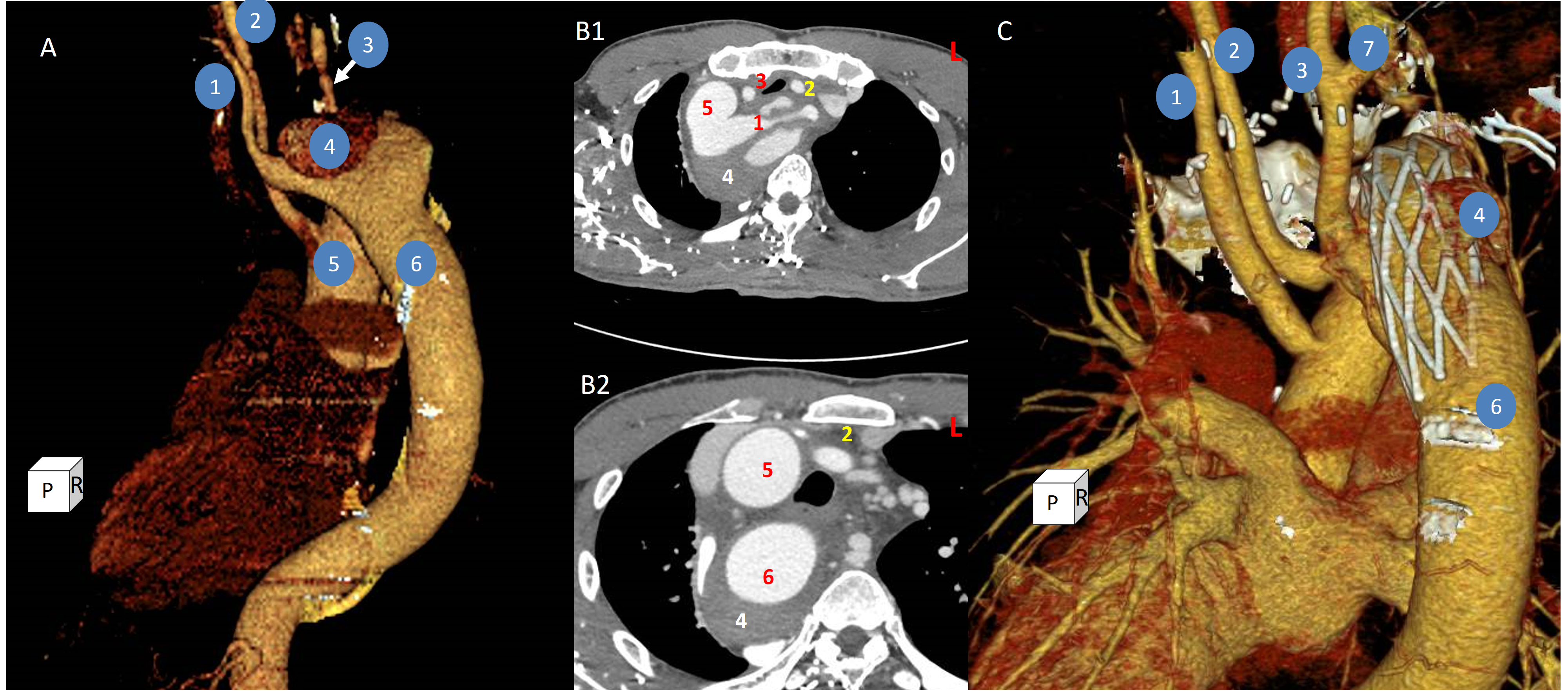

Supplement: Supplementary Figure S2 — CT images of the patient with right arch+ALSA. (A) Pre-operative CT construction image. (B) CT scan images. (C) Postoperative CT construction image. (1) ALSA; (2) LCCA; (3) RCCA; (4) False lumen; (5) Right arch; (6) Right-sided descending thoracic aorta; (7) RSA. [file Image_2.TIF]
