# Supplementary material for: Surgical treatment strategies for patients with type A aortic dissection involving arch anomalies
Source: Front Cardiovasc Med. 2022 Sep 13;9:979431. doi: 10.3389/fcvm.2022.979431 (PMC9513207; doi:10.3389/fcvm.2022.979431)
Supplement: Supplementary file 3 [file Data_Sheet_1.PDF]

Supplemental Material-Surgical procedure

1. Total Arch Replacement(TAR)

1.1 TAR with Four-branched graft

Exposure and cannulation

The procedure is performed by median sternotomy and under the condition of cardiopulmonary bypass(CPB) + selective cerebral perfusion(SCP). Patient lay in supine position and is administered general anesthesia. Arterial blood pressure is monitored in the left radial and left femoral arteries. Cerebral oxymetry is used throughout the procedure to investigate the cerebral blood circulation. In the process of median sternotomy, care should be taken to avoid aortic dissection rupture due to blood pressure changes, especially for patients with acute aortic dissection.

We preferably use right axillary artery cannulation for SCP. The right axillary artery is exposed through an oblique 5 cm incision in the right deltopectoral groove 1 cm below the clavicle. After separating pectoralis major muscle fibers, the pectoralis minor muscle is exposed and retracted to the opposite side with a pull hook. The adjacent veins and brachial plexus are then exposed and carefully mobilized. Arterial line is bifurcated in order to perfuse the brain through the right axillary artery and the descending abdominal aorta through the fourth perfusion side branch of the four-branched graft. In most cases, the right axillary artery can provide adequate perfusion to both cerebral hemispheres through the Circle of Willis. Some patients with arch anomalies can not establish SCP through right axillary artery, especially in patients with aberrant right subclavian artery(ARSA). Other arterial cannulation locations include femoral artery, aortic arch and supra-arch vessels. Other cannulae for CPB include a two-staged venous cannula via right atrium and a left heart vent placed through the right superior pulmonary vein.

After the establishment of CPB, the innominate vein is mobilized and, in some cases, directly dissected for better exposure. The supra-arch vessels are fully exposed and mobilized. After heparinization, CPB is initiated at a flow rate of 2.0-2.4 L/m^2 /min and systemic cooling started to induce hypothermia. The left phrenic nerve and left vagus nerve are gently stripped from the anterior surface of the aorta and care should be taken to protect them. The distal ascending aorta is cross-clamped, then, the ascending aorta is incised and perfused with cold blood cardioplegia via the coronary ostia. During the cooling stage, surgical operations on the aortic root, such as Bentall, David, Yacoub procedures can be performed if needed, and other concomitant procedures can also be done at this time. When the nasopharyngeal temperature reaches about 25°C, the patient is put in a Trendelenburg position at a tilt of about 20° and circulation arrest is started. After three supra-arch vessels are cross-clamped, antegrade SCP

through the right axillary artery is started with a flow of 5-10 mL/kg/min. For patients who are unable to undergo SCP through the right axillary artery, unilateral/bilateral SCP can be achieved through the cannulation of the left/right common carotid artery or left+right common carotid arteries.Radial artery pressure measurement of 20 mmHg or higher during SCP is considered adequate for cerebral perfusion.

Stented elephant trunk placement

Under the condition of hypothermia and circulation arrest(HCA)+SCP, the ascending aorta and aortic arch are dissected longitudinally, extending to a location between the left carotid artery and the left subclavian artery, where the descending aorta is transected. Care should be taken to protect the vagus nerve located on the surface of the distal arch. The supra-arch vessels are transected from the their origins. The stented graft is inserted into the true lumen of the descending aorta, and deployed to compress the false lumen. The delivery system consists of a grip handle, a guide wire and a pull cord with a 2-0 silk thread. During deployment, one hand grasps the handle and the other hand pulls out the guide wire and the stented elephant graft will expand automatically.

The stented elephant trunk(SET) implanted in the descending aorta is approximately 10-15 cm long. The exact length will depend on the location of the primary intimal rupture, the diameter of the aneurysm neck, and the patient's body size. For most patients with aortic dissection, this length is sufficient to cover the intimal tear located between the origin of the left subclavian artery(LSA) and the descending aorta at the level of the left atrium. In some patients with descending aortic aneurysms, a longer 12-15 cm stented graft should be used to extend at least 2 cm distally beyond the aneurysmal neck.

To be noted, in patients with acute dissection, due to the fragility of the aortic wall, great care must be taken during SET insertion to avoid creating new intimal tears. Our method is to bend the flexible SET and implant it along the curvature of the arch and descending aorta so that it fits well with the true lumen morphology.

We use 24-28 mm stented grafts for most aortic dissection cases. However, in patients with aneurysms, the diameter of the stented graft should be at least 15-20% larger than that of the distal neck.

The distal aortic arch is trimmed, and the stented graft is anastomosed to the four-branched vessel graft (Maquene or Terumo) in an end-to-end fashion using 5-0 polypropylene without pledgets. In most cases, this anastomosis is located between the left common carotid artery(LCCA) and LSA; this is a more superficial site for LSA and is easier to suture and hemostasis. The stented graft, the original aortic wall, and the four branched vessel graft should be anastomosed together simultaneously. After the distal anastomosis is completed, the four-branched vessel graft is clamped, and with adequate de-airing, lower body perfusion is restored through the four-branched

vessel graft or femoral artery cannulation, and CPB blood flow is gradually restored to 2.0-2.4 L/m^2/min. During CPB, a mean perfusion pressure should be maintained at 40-60 mmHg.

Arch reconstruction

After SET implantation and its distal anastomosis with the four branched vessel graft, the arch reconstruction will be performed under the condition of SCP and restoration of the lower body perfusion.

Specific arch reconstruction strategies are tailored to different variation of the arch branching patterns.

For patients with ARSA: Figure 2-A/B/C/D shows different arch reconstruction strategy with four-branched vessel graft.

Figure 2-A: Before CPB, the ARSA is fully mobilized and dissected, its distal part is then anastomosed to the right common carotid artery(RCCA) in an end-to-side fashion with 5-0 polypropylene, so as to create a normal anatomy structure like innominate artery. Then, CPB is established via routine right axillary artery cannulation and right atrial intubation. During the arch reconstruction, the LCCA is anastomosed to the 8 mm side branch of the four-branched vessel graft end-to-end with running stitches of 5-0 or 6-0 polypropylene. Upon completion of the anastomosis, the clamp of LCCA is withdrawn to achieve bilateral SCP, and then, LSA and RCCA are attached to other 2 side branches of the four-branched graft in an end-to-end fashion.

Figure 2-B: Extra-anatomic revascularization of ARSA. Subsequently to the establishment of SCP and restoration of the lower body perfusion, the following procedure are proceeded sequentially: The LCCA、LSA and RCCA are sequentially anastomosed to the side branches of the four-branched graft en-to-end, proximal end-to-end anastomosis in the ascending aorta, remove the cross-clamp to restore cardiac perfusion, after the heart resumes beating, the fourth perfusion side branch graft is anastomosed to the right axillary artery in an end-to-side fashion through the right thoracic cavity and the second intercostal space, to achieve an extra-anatomic revascularization of ARSA.

Figure 2-C: SCP is achieved by cannulation of LCCA. End-to-end anastomosis of RCCA, LSA, and LCCA with 3 side branches are completed sequentially. Then, the distal ARSA is anastomosed to the right common carotid artery’s corresponding side branch.

Figure 2-D: SCP is achieved by cannulation of LCCA. RCCA, distal ARSA, and LCCA are anastomosed to 3 side branches of the four-branched vessel graft in sequence, and then, the LSA is anastomosed to the corresponding side branch of the LCCA end-to-side with a extended artificial vessel.

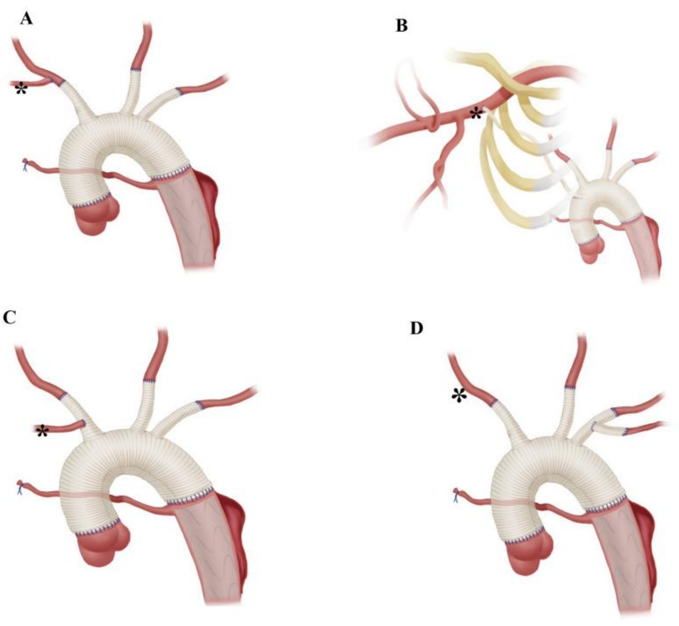

Figure 2:Arch technique for ARSA in patients with type A aortic dissection.

**: Distal ARSA; A: staged TAR for ARSA; B: extra-anatomic revascularization of the right axillary artery; C, ARSA connected to the vessel graft of the RCCA; D,ARSA directly anastomosed to the 8mm arch branch of the 4-branched graft;*

For patients with Bovine: Figure 3-A/B shows different arch management strategy with four-branched graft. Unilateral or bilateral SCP is achieved by cannulation of the right axillary artery. The LCCA (3-A) or proximal innominate artery (3-B) is anastomosed to one of the side branches end-to-end firstly, subsequently, the remaining supra-arch vessel(s) is/are anastomosed to the remaining side branch(es). Patients whose LCCA and innominate artery(IA) a share the same origin usually underwent procedure of figure 3-A, patients whose LCCA originate from IA usually underwent procedure of figure 3-B.

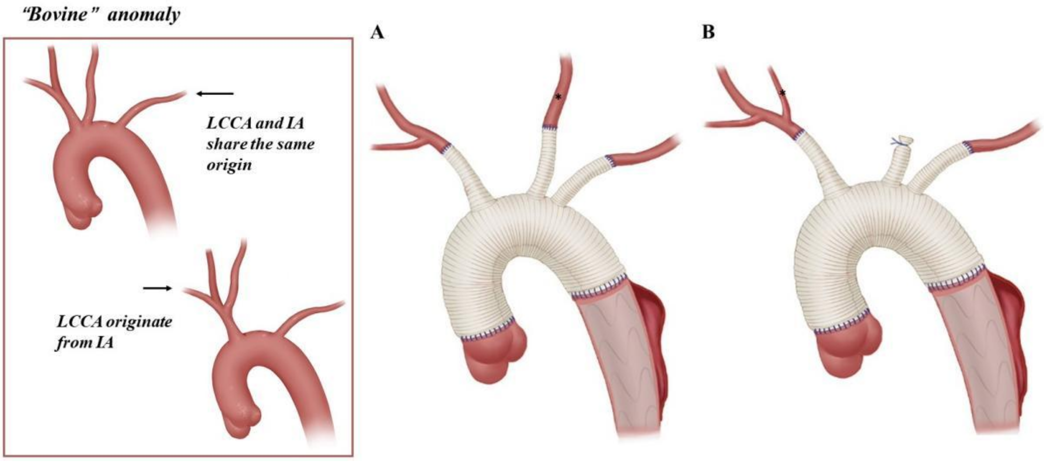

Figure 3 :Arch technique for 'bovine' anomaly in patients with type A aortic dissection.

A, Reconstruction with 3 graft branches for 'bovine' anomaly; B, Reconstruction with 2 graft branches for ' bovine' anomaly

For patients with ILVA: Figure 4-A/B shows different arch reconstruction strategy with four-branched graft. Firstly, the LCCA is anastomosed to the 8 mm side branch end-to-end and by adequate de-airing and removing the clamp of LCCA, bilateral SCP is achieved. Then, LSA and IA are sequentially anastomosed to the side branches of the four-branched graft in end-to-end manners. Finally, the vertebral artery is anastomosed to the LSA(4-A) or its corresponding side branch graft(4-B) in an end-to-side fashion with 5-0 or 6-0 polypropylene.

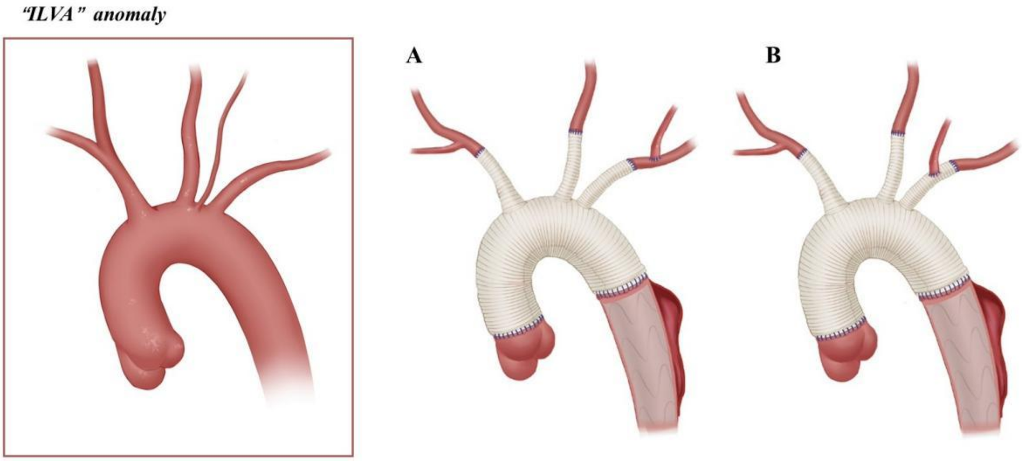

Figure 4 :Arch technique for 'ILVA' anomaly in patients with type A aortic dissection.

A, ILVA anastomosed to the LSA; B, ILVA anastomosed to the LSA's graft branch

A patient with right sided arch combining aberrant left subclavian artery (ALSA) malformation underwent TAR with a four-branched graft, and his arch management strategy is shown in Figure 5. Under the condition of SCP with cannulation in the RCCA, LCCA is transected and anastomosed to the 8mm side branch, then, this

branch is fully de-aired. The ALSA and RCCA are trimmed and sutured to the 8 and 10 mm side branches of the 4-branched graft in an end-to-end fashion with running stitches of 5-0 polypropylene, right after, the RSA is anastomosed to the RCCA in an end-to-side fashion.

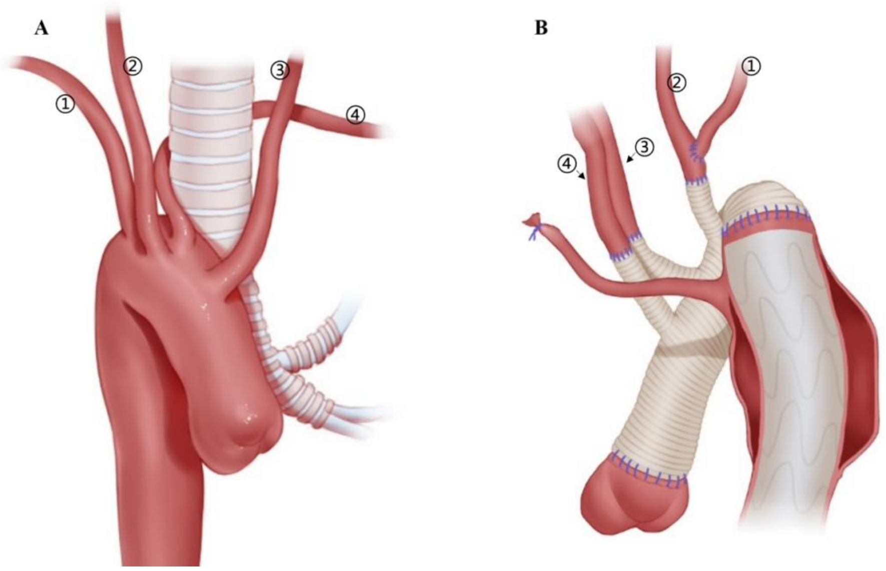

Figure 5: Arch technique for patients with right arch and ALSA

1, RSA; 2, RCCA; 3, LCCA; 4, ALSA; A, Schematic diagram of the anatomy of right arch + ALSA, from the front view. B, Arch technique for patient with right arch + ALSA, from the posterior view. ALSA, from the posterior view.

Proximal procedure.

After completion of the arch reconstruction, the four-branched graft is anastomosed to the ascending aortic graft or ascending aortic wall tissue in an end-to-end fashion. After the ascending aorta is completely de-aired, the cross clamp is removed, coronary perfusion is restored and heart resume beating. Sometimes, to reduce the duration of myocardial ischemia, the proximal anastomosis of the ascending aorta can be done right after the completion of the first anastomosis of the supra arch vessels (usually LCCA or IA). This will allow bilateral SCP and less myocardial ischemia time. After the completion of the proximal anastomosis, the coronary perfusion will be recovered, and then, the remaining supra-arch vessels will be repaired during the rewarming phase.

For better hemostasis, we sometimes create a Cabrol shunt from the perivascular space of the artificial vessel to the right atrium by using the remnant aortic wall tissue and a piece of bovine pericardial patch, so that any postoperative bleeding will not be a problem.

Hemostasis and chest closure is performed in a routine manner.

1.2 TAR with En bloc technique

Exposure and cannulation

The exposure process is the same as described above.

Femoral artery is our preferred arterial cannulation place for CPB. It can be found in the middle and inner 1/3 area 1cm below the groin. CPB is accomplished via femoral cannulation and right atrial intubation.After full mobilization of the supra-arch vessels and systemic heparinization, CPB is initiated at a flow rate of 2.0-2.4 L/m2/min. The supra-arch vessels are then clamped, SCP is achieved via cannulation of the right axillary artery or supra-arch vessels with a flow of 5-10 mL/kg/min. Under the condition of HCA and SCP, The anterior wall of the aortic arch is incised longitudinally. No dissection in the supra-arch vessels is found intraoperatively.

Arch reconstruction

En bloc technique is a relatively simple arch reconstruction method. After insertion of the SET, a blocking balloon is placed inside it and then the femoral cannula is used to perfuse the lower half of the body. The gap between the entrance of the descending aorta and SET is reinforced with running stitches of 5-0 polypropylene, and then the stent-free sewing edge (5 cm long Dacron graft) of the SET is trimmed and sutured to the native aortic wall containing the origins of the supra-arch vessels (shown in Figure 2-E) with 4-0 or 5-0 polypropylene.

The ARSA is dissected and ligated, and after chest closure, RCCA-to-RSA bypass using cervical and supraclavicular fossa incisions is performed, a single 7-mm GORE-TEX graft is used and anastomosed to the RCCA and RSA in end-to-side manners with 7-0 GORE-TEX sutures.

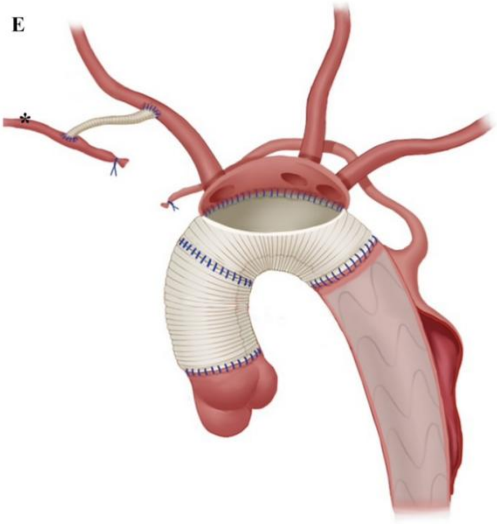

Figure 2-E, En bloc technique for ARSA;

2 Right hemi-arch replacement

Exposure and cannulation

Under general anesthesia and median sternotomy, CPB is established by right femoral arterial cannulation and right atrial intubation. After careful mobilization of the ascending aorta, aortic arch and supra-aortic vessels, the distal ascending aorta is cross-clamped and the ascending aorta is incised and perfused with cold blood cardioplegia via the coronary ostia. Concomitant cardiac procedures, such as valve replacement, sinus reconstruction, can be performed during the cooling phase.

Arch reconstruction

The nasopharyngeal temperature should be reduced to 20-25°C. Unilateral or bilateral SCP is achieved by cannulation of the IA (Patients with Bovine anomaly). Under the condition of HCA, the aortic arch is incised along the lateral side of the lesser curvature of the aortic arch. Surgical probe prove that there is no involvement of the supra-arch vessels or descending aorta, and dissection only involve the lesser curvature of the aortic arch.

A blocking balloon is placed in the descending aorta, and then recover the femoral cannula perfusion to the lower body. The lesser curvature of the aortic arch is trimmed to facilitate anastomosis. The right hemi-arch replacement is done with a single Dacron graft vessel, it is anastomosed to the trimmed margin of the lesser curvature of the aortic arch in an end-to-end manner with running stitches of 5-0 polypropylene. Upon completion of the anastomosis, the blocking balloon is removed and the proximal Dacron graft is fully de-aired and cross clamped, so that the upper extremity can be perfused by the femoral cannula. Then, the Dacron graft is anastomosed to the proximal ascending aorta, subsequently, the cross clamp is removed and coronary perfusion is restored.

Sometimes, to reduce the duration of myocardial ischemia, the proximal anastomosis of the Dacron graft and ascending aorta can be done during the cooling phase. By doing this, before the completion of the distal anastomosis, the blocking balloon should be removed and the Dacron graft should be fully de-aired by retrograde perfusion of the femoral cannula.

3 Hybrid techniques

Extra-anatomic revascularization

General anesthesia is administered, patient is put in a horizontal position, the cervical and supraclavicular fossa regions are our preferred location for surgical probe. RCCA and LCCA can be exposed by a transverse cervical incision, RSA and LSA can be exposed in the supraclavicular fossa area, with attention to protect the brachial plexus nerve and vagus nerve.After heparinization, guide the GORE-TEX graft through the sternocleidomastoid muscle and the posterior aspect of the internal jugular vein, then, carotid-to-subclavian bypass are accomplished based on the lesion anatomy feature. 7-mm GORE-TEX graft is anastomosed to the subclavian artery and common carotid artery in end-to-side manners, with running stitches of 7-0 GORE-TEX sutures. Once the completion of the anastomosis the proximal subclavian artery(ies) should be ligated (Figure 2-F shows).

TEVAR

After completing the extra-anatomic revascularization, the right femoral artery is openly exposed, a 6F arterial sheath is inserted, and a 6F pigtail catheter is placed through the arterial sheath, and the pigtail catheter is proved to be located in the true lumen by angiography at the level of the first lumbar vertebra, diaphragm, and ascending aorta, respectively. A rigid guidewire is placed through the pigtail catheter. The thoracic stent graft is deployed and released retrograde via femoral artery access with the guide of the rigid guidewire. It is generally oversized by 10-15% according to the arch pathologies. After the thoracic stent graft introduction upward into the aortic arch, angiography confirm it is in the accurate deployment position.

A successful hybrid procedure is defined as accurate deployment of thoracic stent graft and patency of the extra-anatomic bypass confirmed by angiography.The thoracic stent graft needs to cover the origin of ARSA and primary intimal tear(s). A proximal landing zone of at least 2 cm of the healthy aortic wall should be considered in this process.

F

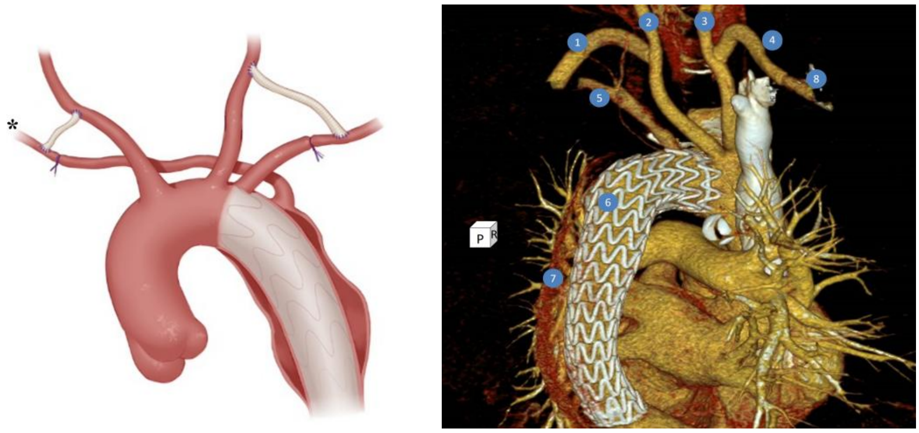

F, Hybrid technique for ARSA
